# Evidence of a distinct peripheral inflammatory profile in sport-related concussion

**DOI:** 10.1186/s12974-019-1402-y

**Published:** 2019-01-26

**Authors:** Alex P. Di Battista, Nathan Churchill, Shawn G. Rhind, Doug Richards, Michael G. Hutchison

**Affiliations:** 1 Faculty of Kinesiology & Physical Education, University of Toronto, Toronto, ON Canada; 2 Defence Research and Development Canada, Toronto Research Centre, Toronto, ON Canada; 3 Neuroscience Program, Keenan Research Centre for Biomedical Science of St. Michael’s Hospital, Toronto, ON Canada; 4 Keenan Research Centre for Biomedical Science of St. Michael’s Hospital, Toronto, ON Canada; 5 David L. MacIntosh Sport Medicine Clinic, Faculty of Kinesiology & Physical Education, University of Toronto, Toronto, ON Canada

**Keywords:** Inflammation, Concussion, Chemokines, Cytokines, Biomarkers

## Abstract

**Background:**

Inflammation is considered a hallmark of concussion pathophysiology in experimental models, yet is understudied in human injury. Despite the growing use of blood biomarkers in concussion, inflammatory biomarkers have not been well characterized. Furthermore, it is unclear if the systemic inflammatory response to concussion differs from that of musculoskeletal injury. The purpose of this paper was to characterize systemic inflammation after injury in athletes with sport-related concussion or musculoskeletal injury.

**Methods:**

A prospective, observational cohort study was conducted employing 175 interuniversity athletes (sport-related concussion, *n* = 43; musculoskeletal injury, *n* = 30; healthy, *n* = 102) from 12 sports at a sports medicine clinic at an academic institution.

High-sensitivity immunoassay was used to evaluate 20 inflammatory biomarkers in the peripheral blood of athletes within 7 days of injury (subacute) and at medical clearance. Healthy athletes were sampled prior to the start of their competitive season. Partial least squares regression analyses were used to identify salient biomarker contributions to class separation between injured and healthy athletes, as well as to evaluate the relationship between biomarkers and days to recovery in injured athletes.

**Results:**

In the subacute period after injury, compared to healthy athletes, athletes with sport-related concussion had higher levels of the chemokines’ monocyte chemoattractant protein-4 (*p* < 0.001) and macrophage inflammatory protein-1β (*p* = 0.001); athletes with musculoskeletal injury had higher levels of thymus and activation-regulated chemokine (*p* = 0.001). No significant differences in biomarker profiles were observed at medical clearance. Furthermore, concentrations of monocyte chemoattractant protein-1 (*p* = 0.007) and monocyte chemoattractant protein-4 (*p* < 0.001) at the subacute time point were positively correlated with days to recovery in athletes with sport-related concussion, while thymus and activation-regulated chemokine was (*p* = 0.001) positively correlated with days to recovery in athletes with musculoskeletal injury.

**Conclusion:**

Sport-related concussion is associated with perturbations to systemic inflammatory chemokines that differ from those observed in athletes with a musculoskeletal injury. These results support inflammation as an important facet of secondary injury after sport-related concussion that can be measured systemically in a human model of injury.

**Electronic supplementary material:**

The online version of this article (10.1186/s12974-019-1402-y) contains supplementary material, which is available to authorized users.

## Introduction

Sport-related concussion (SRC) is a traumatic brain injury (TBI) induced by biomechanical forces. It is considered a mild form of TBI since no abnormalities are identified on standard structural neuroimaging [1]. The injury can result in a constellation of symptoms relating to impaired cognition, balance, vision, physical and emotional health, and sleep disturbances [2, 3]. Full symptom resolution varies from days to weeks [1, 4, 5], with a number of patients (~ 15%) experiencing prolonged symptoms that can last months to years after injury [1]. Historically, a fundamental lack of understanding of the biological sequelae following injury has impeded the clinical translation of treatment options for SRC.

Recently, studies employing advanced neuroimaging and fluid biomarkers have enhanced our knowledge of the mechanistic underpinnings of SRC in humans. These investigations have yielded evidence to support acute and subacute structural and functional alterations in the brain, including changes to functional connectivity [6–8] and cerebral blood flow [7, 9], axonal/neuronal injury [10–13], oxidative damage/stress [14], and inflammation [7, 15, 16]. In a number of cases, perturbations have also been identified at medical clearance [6, 7, 14, 17], suggesting a possible disconnect between clinical and biological recovery. However, despite being a central and well-studied component of secondary injury in moderate and severe traumatic brain injury (TBI) [18–21], the involvement of inflammation in SRC, particularly in the acute and subacute phases after injury, is comparatively less well characterized [15, 16]. Elucidating its role has important implications for characterizing the recovery process, as well as expanding potential therapeutic strategies.

Blood biomarkers are useful tools to measure inflammatory perturbations across the injury severity spectrum of TBI [14, 18, 19, 22, 23], and due to their relatively non-invasive and cost-effective nature, allow for sampling in a large number of participants. While characterizing inflammation in the peripheral blood can help uncover the potential systemic consequences of brain injury [24–27], because of the significant gene expression overlap between the peripheral blood and the CNS [28], it may also be used to reveal the inflammatory status of the brain. In view of this, a small number of studies have shown that SRC results in perturbations to systemically measured inflammatory indices. For example, we recently found a relationship between decreased global connectivity and higher peripheral blood levels of the chemokines' monocyte chemoattractant protein (MCP)-1 and MCP-4 in athletes within the first week of concussion [7]. In addition, there is evidence that inflammatory gene expression in peripheral blood mononuclear cells is altered within 6 h and at 7 days post-injury [15, 16], and there are recent preliminary findings of increased blood concentrations of brain-derived extracellular vesicles carrying tumor necrosis factor (TNF)-α and interleukin (IL)-8 in the days following SRC [29]. Yet, despite the promise of utilizing peripheral blood biomarkers of inflammation to study human brain injury, prototypical inflammatory correlates—cytokines and chemokines—have not been well characterized throughout recovery following SRC.

Given the complex, pleiotropic nature of the immune response to injury, an attempt at representing the inflammatory response to SRC need be mindful of potential confounds. First, the immune response differs between males and females [30]. Second, athletes are often injured during physical activity; vigorous exercise has known effects on the peripheral inflammatory response [31–34]. Lastly, in an athletic population it is important to assess the inflammatory response to musculoskeletal (MSK) injury in order to determine whether perturbations seen after SRC are specific to head trauma, or represent a general inflammatory response to injury [35–39].

Hence, the purpose of this study was to characterize inflammatory cytokine and chemokine profiles in the peripheral blood of athletes after SRC or MSK injury, both within the first week after injury and at medical clearance. We hypothesized that SRC would be associated with a unique blood signature distinct from MSK injuries, with persistent alterations present at medical clearance.

## Methods

### Participants

This prospective, observational cohort study enrolled 175 athletes (male (m), *n* = 92; female (f), *n* = 83), recruited from 12 interuniversity sport teams at a single institution, including basketball (m and f), field hockey (f), football (m), ice hockey (m and f), lacrosse (m and f), mountain biking (m), rugby (m and f), soccer (m and f), swimming (f), track and field (m and f), volleyball (m and f), and water polo (m) between 2014 and 2018. Forty-three athletes were recruited following the diagnosis of a SRC (median = 4, range = 3-5). Both the diagnosis and medical clearance to return-to-play (RTP) were made by a staff physician at a single sport medicine clinic in accordance with the Concussion in Sport Group guidelines [1]. Briefly, medical clearance to RTP included the evaluation of symptom status, balance, and cognitive abilities following the successful completion of a graded exercise protocol. In addition, 30 athletes were recruited following a MSK injury (median = 4, range = 3–6). MSK injury was defined as an injury to the structure and/or function of the musculoskeletal system requiring an athlete to abstain from all sport participation for a minimum of 7 days. MSK injuries were also diagnosed by staff physicians, and RTP was determined by a primary care sports and exercise medicine physician who evaluated athletes based on restoration of sport-specific function to the injured part, as well as psychosocial readiness and risk of re-injury. Finally, 102 healthy athletes were recruited prior to the beginning of their athletic season. For those athletes with a history of SRC, those who had been diagnosed within 6 months were excluded. All study participants provided written informed consent prior to enrollment, and all study procedures were in accordance with the declaration of Helsinki and approved by the Health Sciences Research Ethics Board, University of Toronto (protocol reference # 27958).

### Blood biomarkers

Blood was sampled from injured athletes within 7 days (SRC median = 4, range = 2–7; MSK injury median = 5, range = 2–8). A second blood sample was taken within a median of 5 days of medical clearance to RTP (range = 1–14). Healthy athlete blood samples were acquired prior to the beginning of the athletic season. Blood was not taken from athletes who presented with a known acute infection or illness at the time of sampling or were taking any medications beyond birth control. Venous blood was drawn into a 10-mL K_2_EDTA tube and was equilibrated for approximately 1 h at room temperature before a 2-min centrifugation using a PlasmaPrep 12™ centrifuge (Separation Technology Inc., FL, USA). Plasma supernatant was then aliquoted and frozen at − 70 °C until analysis.

Twenty blood biomarkers were analyzed by immunoassay using Meso Scale Diagnostics 96-well MULTI-SPOT® technology. Nineteen cytokines and chemokines were quantitated using two V-PLEX® assays: Proinflammatory Panel 1 included interferon (IFN)-γ, interleukin (IL)-1β, IL-2, IL-4, IL-6, IL-8, IL-10, IL-12p70, IL-13, and tumor necrosis factor (TNF)-α, and Chemokine Panel 1 included eotaxin, eotaxin-3, interferon gamma-induced protein (IP)-10, monocyte chemoattractant protein (MCP)-1, MCP-4, macrophage-derived chemokine (MDC), macrophage inflammatory protein (MIP)-1α, MCP-1β, and thymus and activation-regulated chemokine (TARC). Myeloperoxidase (MPO) was run as a single-plex assay.

### Symptoms

At the time of blood draw, symptoms were ascertained by a 22-item post-concussion symptom scale using a 7-point Likert rating as part of the sport concussion assessment tool (SCAT). The SCAT is the most widely used tool to assist in the diagnosis, management, and prognosis of individuals with concussion [1]. Symptom severity is obtained by summing the rated symptom score for each symptom. This scale has shown reliability and validity for the assessment of both symptom presence and severity [40, 41].

### Statistical analysis

Preprocessing was performed on all raw biomarker data to arrive at a final dataset of markers for statistical analysis. A useable biomarker value was defined as a value within the detection limits of the assay as described by the manufacturer, displaying a coefficient of variation < 25% between duplicate samples. A biomarker was then included in the final dataset if it had ≥ 80% useable values. For a complete list of biomarker detectability, please see Additional file 1: Table S1, and for technical information on individual biomarker assays, please see Additional file 2: Table S2.

Prior to statistical analysis, biomarker data was checked for violations of normality within each group (SRC, MSK, healthy), as heavy tails can impact correlational analyses. Kurtosis for each biomarker was evaluated against 1000 resamples of a random normal Gaussian model. Biomarker kurtosis in the healthy group ranged from 4.4 (*p* = 0.017) to 43 (*p* < 0.001), in the SRC group from 3.1 (*p* = 0.574) to 12.6 (*p* < 0.001), and in the MSK group from 3.2 (*p* = 0.614) to 14.6 (*p* < 0.001). Hence, all data were rank transformed prior to statistical evaluation.

Between-group comparisons of athlete characteristics found in Table 1 were calculated by bootstrap resampling of the mean difference between groups (5000 iterations) in order to generate a bootstrap ratio (effect size). From this, an empirical *p* value was obtained and corrected at a false discovery rate (FDR) threshold of 0.05.

**Table 1:** Athlete characteristics

Variable	SRC (*n* = 43)	MSK injury (*n* = 30)	Healthy (*n* = 102)
Age (years)	21.2 (18.9–22.1)	20.9 (19.9–22.7)	21.2 (19.6–22.3)
Sex (*n*, % male)	21 (48.8)	17 (56.7)	54 (52.9)
Sport (*n*, %)			
Basketball	2 (4.6)	1 (3.3)	12 (11.8)
Field hockey	2 (4.6)	–	10 (9.8)
Football	6 (13.9)	3 (10.0)	12 (11.8)
Ice hockey	10 (23.2)	8 (26.7)	19 (18.6)
Lacrosse	5 (11.6)	2 (6.7)	11 (10.8)
Mountain biking	1 (2.3)	–	–
Rugby	13 (30.2)	7 (23.3)	11 (10.8)
Soccer	1 (2.3)	3 (10.0)	18 (17.6)
Swimming	–	1 (3.3)	–
Track and field	–	3 (10.0)	–
Volleyball	3 (7.0)	2 (6.7)	8 (7.8)
Water polo	–	–	1 (1)
Symptoms*			
Days to assessment from injury	4 (3–5)	4 (3–6)	–
Total symptoms	9.5 (5.0–14.2)^a,b^	2.0 (1.0–4.2)	2.0. (0–4.0)
Symptom severity	16.0 (5.7–29.7)^a,b^	2.5 (1.0–5.7)	2.0 (0–6.0)
Days to blood sample from injury	4 (range = 2–7)	5 (range = 2–8)	–
Days to medical clearance	25.0 (15.0–55.5)	33.5 (23.5–57.2)	–
Concussion history (*n*, %)	25 (58.1)	14 (46.7)	55 (55.0)**
Time since last concussion (years)	3.2 (1.5–6.6)	4.1 (2.4–4.3)	4.0 (2.7–5.7)

All values are reported as the median and interquartile range, unless otherwise stated

*SRC* sport-related concussion, *MSK* musculoskeletal, *SCAT* sport concussion assessment tool

*Symptom scores at the time of blood draw

**Two subjects did not report on their concussion history and this value was calculated from *n* = 100

^a^FDR corrected at 0.05 vs. healthy controls

^b^FDR corrected at 0.05 vs. MSK injury

The primary aim of this paper was to compare inflammatory biomarker profiles in athletes with SRC or MSK injuries to healthy athletes. However, preliminary analysis in the healthy cohort showed that while time from last bout of physical activity and time from injury to blood-draw were not associated with significant changes in biomarker profiles (Additional file 3: Table S3 and Additional file 4: Figure S1, respectively), multiple biomarker concentrations were significantly higher in males vs. females (Additional file 5: Table S4). Hence, prior to between-group comparisons (SRC vs healthy, MSK injury vs. healthy), biomarker values were transformed into *z*-scores created separately on male and female subjects, and then re-concatenated for further analysis. After the creation of sex-controlled *z*-scores for each comparison, group differences between healthy athletes and athletes with either SRC or MSK injury were evaluated by partial least squares discriminant analysis (PLSDA) [42]. PLSDA is used to identify correlations between multiple predictor variables (biomarkers) and a single binary response variable (SRC/MSK vs. healthy). Weighted contributions of each predictor variable were calculated by generating a bootstrap ratio after resampling (5000 iterations). Empirical *p* values were then obtained and corrected at a FDR of 0.05. Slight variations in the number of subjects in the healthy group for comparisons to SRC (*n* = 87) and MSK injury (*n* = 95) exist because injured athletes who also had pre-season baseline data were removed from their respective healthy comparison groups after they were injured; this ended up in the removal of 15 subjects in the SRC vs. healthy comparison and seven subjects in the MSK injury vs. healthy comparison.

Correlations between subacute biomarker concentrations and days to recovery in athletes with SRC or MSK injury were assessed using a correlational partial least squares (PLS) [42]. PLS is similar to PLSDA, although the response variable (days to recovery) is continuous. Prior to analysis, for each biomarker, *z*-scores were generated separately for male and female injured athletes (SRC or MSK) using the mean of the corresponding biomarker values in the healthy group. For PLS and PLSDA plots, biomarkers are represented by the mean and standard error of the bootstrapped loadings.

## Results

### Subject characteristics

Athlete characteristics are summarized in Table 1. At the time of blood sampling, athletes with SRC reported a median total symptom score of 9.5 and a median symptom severity of 16; both were significantly higher than athletes with MSK injury (total symptoms, *p* < 0.001; symptom severity, *p* < 0.001) and healthy athletes (total symptoms, *p* < 0.001; symptom severity, *p* < 0.001). While athletes with SRC reported significantly more symptoms across all symptom clusters (cognitive (*p* < 0.001), somatic (*p* < 0.001), emotional (*p* = 0.02), fatigue (*p* = 0.02)) compared to healthy athletes, only cognitive (4.0 vs. 0, *p* < 0.001) and somatic (9.5 vs. 1.0, *p* < 0.001) symptom reporting was higher in athletes with SRC vs. those with MSK injury. The median time from injury to medical clearance in athletes with SRC was 25 days (IQR = 15.0–55.5) and 33.5 days (IQR = 23.5–57.2) in athletes with MSK injury. At medical clearance, 11 SRC athletes and 12 athletes with MSK injury were lost due to attrition. A breakdown of injury types in the MSK injury group can be seen in Table 2.

**Table 2:** MSK injury characteristics

	Shoulder/thorax (*n* = 6; 20.0%)	Wrist (*n* = 1; 3.0%)	Hip/thigh (*n* = 5; 16.7%)	Knee/lower leg (*n* = 6; 20.0%)	Ankle/foot (*n* = 12; 40.0%)
Myotendinous	1	–	5	1	1
Ligamentous	1	–	–	2	8
Contusion	–	–	–	1	–
Fracture	2^a^	1	–	–	3
Tear	1	–	–	2	–
Other	1^b^	–	–	–	–

*MSK* musculoskeletal

^a^Both fractures were accompanied by a separated AC joint; one rib fracture, one clavicle fracture

^b^Participant had both an AC separation and dislocated shoulder

### Inflammatory biomarker profiles in athletes with SRC and MSK injury

**Fig. 1:**
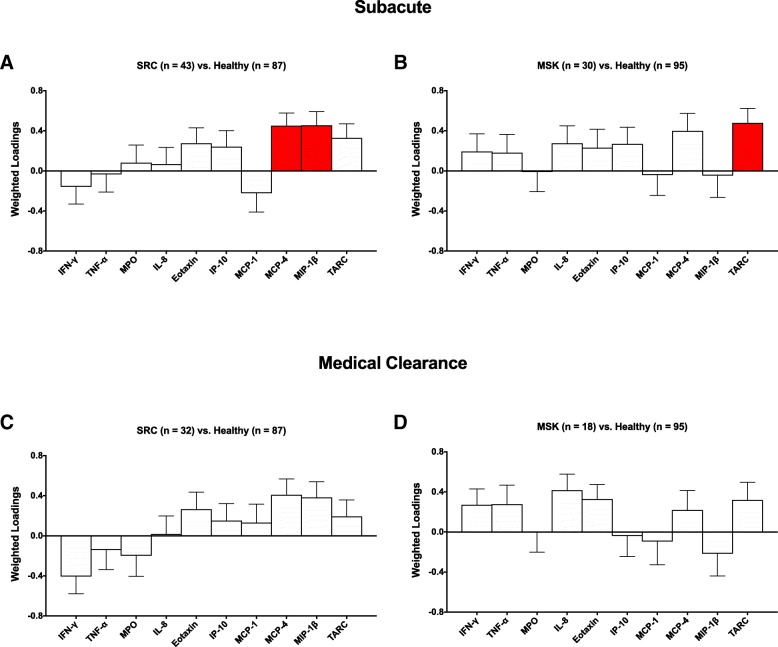
Inflammatory profiles in injured vs. healthy athletes. Interferon (IFN)-γ, tumor necrosis factor (TNF)-α, myeloperoxidase (MPO), interleukin (IL)- 8, eotaxin, interferon gamma-induced protein (IP)-10, monocyte chemoattractant protein (MCP)-1, MCP-4, macrophage inflammatory protein (MIP)-1α, MIP-1β, and thymus and activation-regulated chemokine (TARC). Plots show the contributions of biomarkers towards class separation in the subacute period between **a** athletes with sport-related concussion (SRC) vs. healthy athletes, **b** athletes with musculoskeletal (MSK) injury vs. healthy athletes, and at medical clearance between **c** athletes with SRC vs. healthy athletes and **d** athletes with MSK injury vs. healthy athletes, by partial least squares discriminant analysis (PLSDA). Bars represent biomarker loadings and the standard error derived from bootstrapped resampling (5000 samples). Red bars = significant at a false discovery rate (FDR) < 0.05

PLSDA analyses of inflammatory biomarker profiles in athletes with SRC and MSK injury can be visualized in Fig. 1. In the subacute period after injury, higher concentrations of MCP-4 (bootstrap ratio = 3.5, *p* < 0.001) and MIP-1β (bootstrap ratio = 3.3, *p* = 0.001) significantly contributed to class discrimination between athletes with SRC and healthy athletes (Fig. 1a). Alternatively, higher concentrations of TARC (bootstrap ratio = 3.3, *p* = 0.001) significantly contributed to class discrimination between athletes with MSK injury and healthy athletes (Fig. 1b). At medical clearance, there were no significant biomarker contributions towards class separation between athletes with SRC vs. healthy athletes (Fig. 1c), or athletes with MSK injury vs. healthy athletes (Fig. 1d). Biomarker concentrations in males and females for both the SRC vs. healthy and MSK injury vs. healthy comparisons, at both the acute and medical clearance time points can be found in Tables 3, 4, 5, and 6.

**Table 3:** Biomarker concentrations in athletes with SRC and healthy athletes in the subacute period

Biomarker	Healthy male (*n* = 43)	SRC male (*n* = 21)	Healthy female (*n* = 44)	SRC female (*n* = 22)	*P* value	FDR
IFN-γ	3.5 (2.8–5.2)	3.4 (2.6–5.5)	3.9 (2.9–7.8)	3.7 (2.6–4.8)	0.32	No
TNF-α	1.7 (1.4–2.0)	1.7 (1.4–2.1)	1.5 (1.3–2.0)	1.5 (1.2–1.9)	0.83	No
MPO (ng/mL)	11.2 (8.1–15.8)	12.3 (7.7–20.2)	7.9 (5.8–11.1)	9.0 (7.5–12.2)	0.63	No
IL-8	1.9 (1.6–2.5)	2.0 (1.7–2.4)	1.6 (1.1–2.2)	1.8 (1.0–2.3)	0.69	No
Eotaxin	85.5 (75.1–106.6)	95.4 (83.4–126.7)	73.3 (63.3–89.3)	81.0 (64.5–93.4)	0.07	No
IP-10	159.2 (129.1–226.5)	181.5 (136.1–220.1)	156.1 (132.5–251.2)	207.7 (165.3–288.9)	0.13	No
MCP-1	68.9 (58.8–77.7)	66.9 (57.7–75.6)	58.7 (50.9–69.0)	53.3 (44.2–63.7)	0.25	No
MCP-4	20.8 (16.3–25.5)	26.5 (20.5–32.1)	19.0 (16.8–22.4)	21.2 (18.5–27.7)	0.00	Yes
MIP-1β	29.2 (25.9–40.3)	42.1 (31.4–53.6)	27.7 (22.2–33.6)	31.8 (26.0–39.4)	0.00	Yes
TARC	50.0 (38.9–75.2)	71.3 (44.8–94.6)	42.7 (32.7–54.6)	54.0 (37.5–67.5)	0.02	No

*SRC* sport-related concussion; *FDR* false discovery rate; *IFN-γ* interferon-γ; *TNF-α* tumor necrosis factor-α; *MPO* myeloperoxidase; *IL-8* interleukin-8; *IP-10* interferon gamma-induced protein-10; *MCP-1*, *MCP-4* monocyte chemoattractant protein-1, -4; *MIP-1α*, *MIP-1β* macrophage inflammatory protein-1α, -1β; *TARC* thymus and activation-regulated chemokine

All values reported as the median and interquartile range, in picograms per milliliter unless otherwise stated

*P* values are derived from bootstrap ratios, controlled for sex, corrected at FDR = 0.05

**Table 4:** Biomarker concentrations in athletes with MSK injury and healthy athletes in the subacute period

Biomarker	Healthy male (*n* = 50)	MSK male (*n* = 17)	Healthy female (*n* = 45)	MSK female (*n* = 13)	*P* value	FDR
IFN-γ	3.5 (2.4–4.5)	3.7 (3.2–5.1)	3.9 (2.8–6.6)	4.3 (3.2–9.9)	0.29	No
TNF-α	1.7 (1.4–2.0)	1.9 (1.7–2.2)	1.6 (1.3–2.1)	1.6 (1.2–1.8)	0.36	No
MPO (ng/mL)	10.8 (7.9–16.2)	9.4 (7.9–13.4)	7.2 (5.8–11.0)	8.6 (7.3–10.5)	0.95	No
IL-8	1.9 (1.6–2.5)	2.8 (1.8–3.2)	1.6 (1.2–2.2)	1.9 (1.6–2.2)	0.13	No
Eotaxin	84.2 (73.6–99.3)	98.1 (86.9–117.9)	73.6 (63.8–95.8)	78.0 (57.4–90.2)	0.28	No
IP-10	178.8 (130.2–235.4)	219.3 (153.4–246.6)	155.5 (132.3–246.6)	182.2 (139.1–287.3)	0.10	No
MCP-1	68.8 (60.2–77.4)	72.5 (60.7–80.0)	59.1 (48.8–70.2)	57.5 (49.8–62.1)	0.83	No
MCP-4	21.5 (17.9–25.6)	27.5 (20.6–31.9)	19.0 (16.8–22.4)	22.1 (17.8–27.8)	0.03	No
MIP-1β	35.6 (26.8–42.7)	28.2 (22.7–45.6)	28.2 (23.2–33.6)	31.0 (26.2–34.9)	0.84	No
TARC	49.2 (38.4–75.6)	73.9 (43.2–82.3)	42.9 (33.0–58.1)	58.1 (46.4–70.2)	0.00	Yes

*MSK* musculoskeletal; *FDR* false discovery rate; *IFN-γ* interferon-γ; *TNF-α* tumor necrosis factor-*α*; *MPO* myeloperoxidase; *IL-8* interleukin-8; *IP-10* interferon gamma-induced protein-10; *MCP-1*, *MCP-4* monocyte chemoattractant protein-1, -4; *MIP-1α*, *MIP-*1β macrophage inflammatory protein-1α, -1β; *TARC* thymus and activation-regulated chemokine

All values reported as the median and interquartile range, in picograms per milliliter unless otherwise stated

*P* values are derived from bootstrap ratios, controlled for sex, corrected at FDR = 0.05

**Table 5:** Biomarker concentrations in athletes with MSK injury and healthy athletes at medical clearance

Biomarker	Healthy male (*n* = 43)	SRC male (*n* = 14)	Healthy female (*n* = 44)	SRC female (*n* = 18)	*P* value	FDR
IFN-γ	3.5 (2.8–5.2)	2.9 (2.5–4.3)	3.9 (2.9–7.8)	2.9 (2.3–4.2)	0.02	No
TNF-α	1.7 (1.4–2.0)	1.5 (1.2–1.8)	1.5 (1.3–2.0)	1.6 (1.2–2.1)	0.48	No
MPO (ng/mL)	11.2 (8.1–15.8)	9.0 (5.7–15.2)	7.9 (5.8–11.1)	7.6 (6.0–12.4)	0.34	No
IL-8	1.9 (1.6–2.5)	2.4 (1.5–2.7)	1.6 (1.1–2.2)	1.6 (1.2–1.9)	0.92	No
Eotaxin	85.5 (75.1–106.6)	91.5 (77.4–99.5)	73.3 (63.3–89.3)	87.7 (71.2–95.7)	0.12	No
IP-10	159.2 (129.1–226.5)	177.7 (140.0–201.0)	156.1 (132.5–251.2)	187.2 (145.6–233.3)	0.38	No
MCP-1	68.9 (58.8–77.7)	70.1 (61.0–84.0)	58.7 (50.9–69.0)	57.5 (54.4–78.5)	0.48	No
MCP-4	20.8 (16.3–25.5)	25.2 (23.4–27.1)	19.0 (16.8–22.4)	20.0 (17.8–36.0)	0.00	No
MIP-1β	29.2 (25.9–40.3)	41.3 (36.5–45.0)	27.7 (22.2–33.6)	32.0 (25.0–40.9)	0.01	No
TARC	50.0 (38.9–75.2)	54.3 (45.6–67.3)	42.7 (32.7–54.6)	46.9 (39.1–56.3)	0.24	No

*SRC* sport-related concussion; *FDR* false discovery rate; *IFN-γ* interferon-γ; *TNF-α* tumor necrosis factor-α; *MPO* myeloperoxidase; *IL-8*interleukin-8; *IP-10* interferon gamma-induced protein-10; *MCP-1*, *MCP-4* monocyte chemoattractant protein-1, -4; *MIP-1α*, *MIP-1β* macrophage inflammatory protein-1α, -1β; *TARC* thymus and activation-regulated chemokine

All values reported as the median and interquartile range, in picograms per milliliter unless otherwise stated

*P* values are derived from bootstrap ratios, controlled for sex, corrected at FDR = 0.05

**Table 6:** Biomarker concentrations in athletes with MSK injury and healthy athletes at medical clearance

Biomarker	Healthy male (*n* = 50)	MSK male (*n* = 12)	Healthy female (*n* = 45)	MSK female (*n* = 6)	*P* value	FDR
IFN-γ	3.5 (2.4–4.5)	4.2 (3.6–4.5)	3.9 (2.8–6.6)	3.4 (3.0–4.6)	0.09	No
TNF-α	1.7 (1.4–2.0)	2.1 (1.8–2.2)	1.6 (1.3–2.1)	1.4 (1.2–1.7)	0.14	No
MPO (ng/mL)	10.8 (7.9–16.2)	8.8 (7.2–12.5)	7.2 (5.8–11.0)	9.6 (9.2–14.7)	0.99	No
IL-8	1.9 (1.6–2.5)	2.3 (1.7–3.4)	1.6 (1.2–2.2)	2.4 (1.8–2.7)	0.01	No
Eotaxin	84.2 (73.6–99.3)	92.3 (84.9–107.9)	73.6 (63.8–95.8)	83.4 (70.8–95.0)	0.02	No
IP-10	178.8 (130.2–235.4)	185.4 (151.4–275.9)	155.5 (132.3–246.6)	136.8 (115.9–167.3)	0.85	No
MCP-1	68.8 (60.2–77.4)	65.8 (52.4–85.5)	59.1 (48.8–70.2)	57.3 (49.4–64.5)	0.68	No
MCP-4	21.5 (17.9–25.6)	21.6 (18.6–22.7)	19.0 (16.8–22.4)	28.5 (21.1–30.5)	0.26	No
MIP-1β	35.6 (26.8–42.7)	26.8 (24.5–37.9)	28.2 (23.2–33.6)	26.1 (24.2–37.1)	0.33	No
TARC	49.2 (38.4–75.6)	55.6 (38.7–77.2)	42.9 (33.0–58.1)	88.6 (54.7–112.8)	0.07	No

*MSK* musculoskeletal; *FDR* false discovery rate; *IFN-γ* interferon-γ; *TNF-α* tumor necrosis factor-α; *MPO* myeloperoxidase; *IL-8* interleukin-8; *IP-10* interferon gamma-induced protein-10; *MCP-1*, MCP*-4* monocyte chemoattractant protein-1, -4; *MIP-1α*, *MIP-1β* macrophage inflammatory protein; *TARC* thymus and activation-regulated chemokine

All values reported as the median and interquartile range, in picograms per milliliter unless otherwise stated

*P* values are derived from bootstrap ratios, controlled for sex, corrected at FDR = 0.05

### Correlation between biomarker profiles and days to recovery in SRC and MSK

**Fig. 2:**
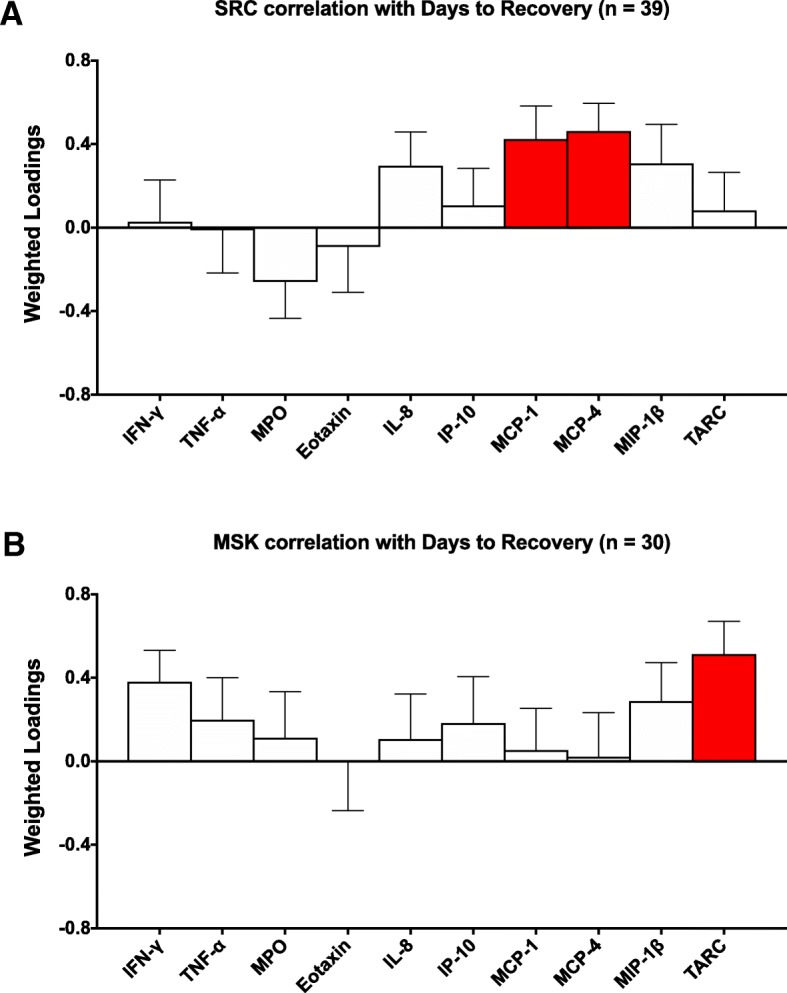
Correlation between inflammatory profiles and days to medical clearance. Interferon (IFN)-γ, tumor necrosis factor (TNF)-α, myeloperoxidase (MPO), interleukin (IL)-8, eotaxin, interferon gamma-induced protein (IP)-10, monocyte chemoattractant protein (MCP)-1, MCP-4, macrophage inflammatory protein (MIP)-1α, MIP-1β, and thymus and activation-regulated chemokine (TARC). Plots show the correlation between biomarkers measured in the subacute period after injury and days to recovery in **a** athletes with a sport-related concussion (SRC) and **b** athletes with a musculoskeletal (MSK) injury, by partial least squares (PLS) analysis. Bars represent biomarker loadings and the standard error derived from bootstrapped resampling (5000 samples). Red bars = significant correlation with days to recovery at a false discovery rate (FDR) < 0.05

PLS analyses depicting the correlation between biomarker values at the subacute time point and days to medical clearance RTP in athletes with SRC or MSK injury can be visualized in Fig. 2. There was a significant positive correlation between days to recovery and both MCP-1 (bootstrap ratio = 2.7, *p* = 0.007) and MCP-4 (bootstrap ratio = 3.5, *p* < 0.001) in athletes with SRC (Fig. 2a). In athletes with MSK, there was a significant positive relationship between days to recovery and TARC (bootstrap = 3.3, *p* = 0.001) (Fig. 2b).

## Discussion

The primary finding of this study was that perturbations to inflammatory biomarker concentrations were detected in the blood of athletes after SRC that differed from those observed in athletes with MSK injury. In both injuries, these differences were observed in the subacute period after injury, but not at medical clearance. Furthermore, we found a significant correlation between inflammatory biomarker concentrations and time to medical clearance in athletes with SRC that differed from the correlation seen in athletes with MSK injury. Our findings suggest that while inflammation appears to be a distinct facet of the natural recovery process after SRC, it may also be linked to pathological sequelae that impede recovery. These findings were observed after controlling for the effects of sex and were not confounded by recent physical activity.

We observed higher blood concentrations of chemokines MIP-1β and MCP-4 in athletes in the subacute period after SRC compared to healthy athletes. This differed from the blood profile seen in athletes with MSK injury, who showed higher concentrations of the T cell chemokine TARC. This is consistent with our previous findings that higher MCP-4 concentrations are correlated with lower functional connectivity in the brain in the subacute period after SRC [7], are supported by preliminary findings of elevations of inflammatory indices in brain-derived extracellular vesicles within the same time period after SRC [29], and are also in line with the increases observed in both aforementioned chemokines acutely after moderate-to-severe TBI [43]. While other studies have observed decreases in inflammatory gene expression in peripheral blood mononuclear cells within a week of SRC [15, 16], there are important disparities between gene and protein expression. Because the source and mechanism underlying the appearance of inflammatory mediators in the blood after SRC is unclear, it is possible that these chemokines may be released from cells/tissues in the periphery or CNS as preformed molecules, not requiring gene transcription [44]. Hence, one hypothesis that requires investigation is that decreased gene expression post-injury may result from negative feedback relating to processes that occur from the time of injury that do not account for the concentration of soluble molecules in the blood. Furthermore, that we found higher concentrations of MIP-1β in athletes with SRC is in line with previous human studies on moderate-to-severe TBI, which have observed increases in systemic [18] and central [45] concentrations of this chemokine in response to injury. However, as MIP-1β has not been studied in sport concussion, its contribution to secondary injury and/or recovery remains undefined.

Contrary to our second hypothesis, we did not find any significant differences in inflammatory profiles between athletes with SRC and healthy athletes at medical clearance, suggesting that for the majority of athletes, inflammation has either resolved or has been tempered by return-to-play. However, as we previously found alterations in blood chemokine concentrations in healthy athletes with a history of multiple concussions (almost 2 years since last concussion) [46], it is possible that inflammation is still a relevant and ongoing process in athletes at medical clearance who have sustained prior concussions. However, the current study was underpowered and hence unable to properly accommodate this sub-group evaluation. Indeed, a preliminary analysis in the current study showed no correlation between the number of prior concussions and biomarker concentrations in athletes after SRC (data not shown), although we did not have the sample size to replicate our earlier study design which separated athletes into three groups: no history of concussion, one previous concussion, and multiple previous concussions [46]. Future investigation is warranted into the potential mediating effect of prior concussion history on the chronicity of inflammation after injury.

Interestingly, we observed a positive correlation between two monocytic chemokines (MCP-1, MCP-4) and days to medical clearance in the subacute period after injury in athletes with SRC, but not in athletes with MSK injury. This suggests that inflammation, beyond its natural role in brain restitution, may be involved in pathological signaling after injury that prolongs recovery. In addition, it may also be possible that higher circulating chemokine levels present prior to injury are detrimental to recovery. Numerous lines of experimental and clinical evidence suggest that chemokines are important components of secondary injury after head trauma [27, 43, 47, 48]. Indeed, we have previously found that elevated levels of MCP-1 in the acute period (< 24 h) after moderate-to-severe TBI are correlated with adverse outcomes and mortality [43]. Furthermore, as previously mentioned, in a recent study we found that MCP-4 was correlated with decreased functional connectivity in the brain in the subacute period after SRC, as well as in athletes with a history of concussion [7]. The latter finding is also supported by another study from our group where we observed elevated concentrations of MCP-4 and MCP-1 in male and female athletes with a history of concussion, respectively [46]. Indeed, experimental evidence has shown a distinct role for MCP-1 in exacerbating damage to brain tissue after injury by promoting macrophage recruitment to the brain and affecting CNS cytokine expression profiles [47, 49]. In these experiments, MCP knockout mice showed decreased brain lesions after injury [47]. While it is difficult to speculate on the translation of these findings from murine to human injury, our results over a series of investigations support a role for MCPs in SRC, with the findings of this investigation suggesting that these chemokines are involved in processes that may impede natural recovery.

While the source(s) of chemokines in the blood after SRC are unknown, experimental evidence from severe murine brain injury suggests that while these molecules may originate in the brain [47, 50], they may also be released systemically from the liver [27, 48, 51, 52]. Indeed, in a review by Catania and colleagues, it was suggested that autonomic centers activated after brain trauma may stimulate the hepatic release of a number of inflammatory mediators, including chemokines, as part of an acute phase response to injury [27]. The authors also state that the peripheral release of chemokines by the liver is an important contributor to the systemic inflammatory response to brain trauma [27]. While this is likely more prominent in the acute phase, experimental evidence has found that the recruitment of peripheral monocytes/macrophages to the brain—a process mediated by monocytic chemokines—may persist for days after injury [53–56]. In humans, evidence of autonomic nervous system (ANS) dysfunction in SRC has been observed previously by our group and others in the subacute period after injury [57, 58], and we have also shown a pathological link between sympathetic activation and inflammation in moderate-to-severe TBI [43]. In view of this, and given the intricate interrelationships between the immune system, hypothalamic-pituitary-adrenal (HPA) axis, and sympathetic nervous system [59–61], our results support others who have identified neuroendocrine and ANS dysfunction after mTBI and concussion [57, 58, 62–65], and suggest that these perturbations may impede recovery. Further research is required to better explain this relationship.

We observed higher concentrations of TARC in athletes with MSK injury compared to healthy athletes, as well as a relationship between TARC and days to recovery. While these findings provide evidence to support a unique systemic inflammatory signature in SRC compared to MSK injury, it is unclear how TARC specifically contributes to the latter. Due to the heterogeneity of injury, it is likely that TARC serves as a general biomarker of an active inflammatory process; inflammation is a known sequela of damage to musculoskeletal tissues [38, 66–68]. However, future studies are necessary to elucidate the pathophysiological role of TARC in MSK injury and how/if it may be used to inform recovery and outcome.

The study must be interpreted in the context of its limitations. While we controlled for sex by generating *z*-scores in injured athletes according to their matched baseline values, this differs from exploring potential differences in the female and male response to SRC. This would require a larger sample size, but is a fruitful endeavor for future research; evidence from animal model experiments has suggested males and females have different inflammatory responses to experimental traumatic brain injury [56, 69], and we have previously reported sex-specific differences in the peripheral blood chemokine profile of athletes with a history of multiple concussions [46]. Furthermore, attrition at medical clearance prevented longitudinal analyses in both the SRC and MSK injury cohorts; in order to preserve power in our analysis at the subacute time point, the data was analyzed cross-sectionally. Lastly, a more acute sampling time point (e.g., within 24 h), while challenging in an athletic population, would have made for a more fulsome evaluation of systemic inflammatory signaling throughout the recovery process. However, despite these limitations, we were able to identify significant differences in inflammatory chemokine concentrations in the blood of athletes with SRC that differed from those observed in athletes with MSK injury, using a robust statistical framework.

## Conclusions

Our findings support distinct perturbations to the inflammatory profile in the blood of athletes in the subacute period after SRC as compared to MSK injury, highlighted by elevated concentrations of chemokines MCP-4 and MIP-1β; these perturbations are attenuated at medical clearance. Furthermore, higher blood concentrations of MCP-1 and MCP-4 in the subacute period after injury are positively correlated with days to recovery in athletes with SRC, but not in athletes with MSK injury, indicating a potentially pathological role for systemic inflammation that may impede recovery. These results support inflammation as a distinct and important component of secondary injury after SRC.

## Additional files

Additional file 1: **Table S1.** Biomarker detectability information. (DOCX 17 kb)

Additional file 2: **Table S2.** Biomarker assay performance information. (DOCX 17 kb)

Additional file 3: **Table S3.** PLSDA analysis of biomarker concentrations in healthy athletes dichotomized into two groups: ≤ 3 h from their last bout of physical activity vs. > 3 h from their last bout of physical activity. (DOCX 16 kb)

Additional file 4: **Figure S1.** PLS correlations between inflammatory profiles and days to blood draw post-injury in athletes with SRC. (DOCX 14 kb)

Additional file 5: **Table S4.** PLSDA analysis of biomarker concentrations in healthy male vs. female athletes. (DOCX 16 kb)

## Abbreviations

ANS: Autonomic nervous system
CNS: Central nervous system
f: Female
FDR: False discovery rate
HPA: Hypothalamic pituitary adrenal
IFN: Interferon
IL: Interleukin
IP: Interferon gamma-induced protein
IQR: Interquartile range
K_2_EDTA: Dipotassium ethylenediaminetetraacetic acid
m: Male
MCP: Monocyte chemoattractant protein
MDC: Macrophage-derived chemokine
MIP: Macrophage inflammatory protein
MPO: Myeloperoxidase
MSK: Musculoskeletal
mTBI: Mild traumatic brain injury
PLS: Partial least squares
PLSDA: Partial least squares discriminant analysis
RTP: Return-to-play
SCAT: Sport concussion assessment tool
SRC: Sport-related concussion
TARC: Thymus and activation-regulated chemokine
TBI: Traumatic brain injury
TNF: Tumor necrosis factor
