# Supplementary material for: Evidence of a distinct peripheral inflammatory profile in sport-related concussion
Source: J Neuroinflammation. 2019 Jan 26;16:17. doi: 10.1186/s12974-019-1402-y (PMC6347801; doi:10.1186/s12974-019-1402-y)
Supplement: Supplementary file 1 — Table S1. Biomarker detectability information. (DOCX 17 kb) [file 12974_2019_1402_MOESM1_ESM.docx]

**Additional file 1: Table S1**. Biomarker Detectability

|  | **Detectable Samples – n (%)** | | |
| --- | --- | --- | --- |
| **Marker** | **Healthy (n = 102)** | **SRC (n = 43)** | **MSK (n = 30)** |
| IFN-γ | 100 (98) | 39 (90.7) | 29 (96.7) |
| TNF-α | 102 (100) | 43 (100) | 30 (100) |
| IL-1β | 4 (3.9) | 4 (9.3) | 3 (10.0) |
| IL-2 | 24 (23.5) | 11 (25.6) | 7 (23.3) |
| IL-4 | 1 (1.0) | 2 (4.65.0) | 0 (0) |
| IL-6 | 43 (42.2) | 16 (37.2) | 16 (53.3) |
| IL-8 | 102 (100) | 43 (100) | 30 (100) |
| IL-10 | 75 (73.5) | 27 (62.8) | 21 (70.0) |
| IL-12 p70 | 14 (13.7) | 9 (20.9) | 4 (13.3) |
| IL-13 | 6 (5.9) | 3 (7.0) | 3 (10.0) |
| MPO | 99 (97.1) | 40 (93.0) | 29 (96.7) |
| Eotaxin | 102 (100) | 41 (95.3) | 30 (100) |
| Eotaxin-3 | 56 (54.9) | 32 (74.4) | 24 (80.0) |
| IP-10 | 101 (99.0) | 43 (100) | 28 (93.3) |
| MCP-1 | 89 (87.3) | 43 (100) | 30 (100) |
| MCP-4 | 96 (94.1) | 41 (95.3) | 29 (96.7) |
| MDC | 50 (49.0) | 22 (51.2) | 18 (60.0) |
| MIP-1α | 41 (40.2) | 19 (44.2) | 14 (46.7) |
| MIP-1β | 95 (93.1) | 43 (100) | 30 (100) |
| TARC | 96 (94.1) | 41 (95.3) | 28 (93.3) |

sport-related concussion (SRC); musculoskeletal injury (MSK); interferon (IFN)-γ; tumor necrosis factor (TNF)-α; interleukin (IL)-1β, -2, -4, -6, -8, -10, -12p70, -13; myeloperoxidase (MPO); interferon gamma-induced protein (IP)-10; monocyte chemoattractant protein (MCP)-1, -4; macrophage-derived chemokine (MDC); macrophage inflammatory protein (MIP)-1α, -1β; thymus and activation-regulated chemokine (TARC).
