# Supplementary material for: Evidence of a distinct peripheral inflammatory profile in sport-related concussion
Source: J Neuroinflammation. 2019 Jan 26;16:17. doi: 10.1186/s12974-019-1402-y (PMC6347801; doi:10.1186/s12974-019-1402-y)
Supplement: Supplementary file 2 — Table S2. Biomarker assay performance information. (DOCX 17 kb) [file 12974_2019_1402_MOESM2_ESM.docx]

**Additional file 2: Table S2**. Biomarker assay information

| **Marker** | **CV >25%** | **Outside LOD** | **Useable** | **Intraassay CV** | **Assay LLOD** |
| --- | --- | --- | --- | --- | --- |
| IFN-γ | 2.6 | 3.4 | 94.0 | 8.1 | 0.37 (0.21 – 0.62) |
| TNF-α | 0.3 | 0.2 | 99.5 | 7.4 | 0.04 (0.01 – 0.13) |
| IL-1β | 1.8 | 89.8 | 8.4 | 10.0 | 0.05 (0.01 – 0.17) |
| IL-2 | 3.9 | 72.8 | 23.2 | 9.8 | 0.09 (0.01 – 0.29) |
| IL-4 | 0.5 | 98.5 | 1.0 | 14.1 | 0.02 (0.01 – 0.03) |
| IL-6 | 3.9 | 53.5 | 42.6 | 9.2 | 0.06 (0.05 – 0.09) |
| IL-8 | 0.6 | 0 | 99.3 | 5.9 | 0.07 (0.03 – 0.14) |
| IL-10 | 8.9 | 22.3 | 68.8 | 10.2 | 0.04 (0.02 – 0.08) |
| IL-12p70 | 2.8 | 80.0 | 17.2 | 9.4 | 0.11 (0.02 – 0.89) |
| IL-13 | 0.2 | 91.4 | 8.4 | 9.2 | 0.24 (0.03 – 0.73) |
| MPO | 3.2 | 0 | 97.2 | 8.5 | 33.0 (NA) |
| Eotaxin | 1.5 | 0 | 98.5 | 5.9 | 3.26 (2.41 – 5.12) |
| Eotaxin-3 | 11.4 | 20.6 | 68.0 | 9.9 | 1.77 (1.29 – 4.13) |
| IP-10 | 0.5 | 0.6 | 98.9 | 5.9 | 0.37 (0.22 – 0.72) |
| MCP-1 | 0.2 | 5.8 | 94.0 | 5.2 | 0.09 (0.06 – 0.31) |
| MCP-4 | 0.5 | 5.7 | 93.8 | 7.0 | 1.69 (1.60 – 1.75) |
| MDC | 1.6 | 37.6 | 60.6 | 7.2 | 1.22 (1.14 – 1.26) |
| MIP-1α | 6.5 | 49.4 | 44.1 | 9.5 | 3.02 (2.28 – 4.01) |
| MIP-1β | 0.2 | 3.7 | 96.1 | 5.1 | 0.17 (0.08 – 0.32) |
| TARC | 4.1 | 3.4 | 92.5 | 5.7 | 0.22 (0.17 – 0.54) |

coefficient of variation (CV); limit of detection (LOD); lower limit of detection (LLOD);

interferon (IFN)-γ; tumor necrosis factor (TNF)-α; interleukin (IL)-1β, -2, -4, -6, -8, -10, -12p70, -13; myeloperoxidase (MPO); interferon gamma-induced protein (IP)-10; monocyte chemoattractant protein (MCP)-1, -4; macrophage-derived chemokine (MDC); macrophage inflammatory protein (MIP)-1α, -1β; thymus and activation-regulated chemokine (TARC).

All values reported as percent, other than Assay LLOD, which is reported as the median and range.
