# Supplementary material for: Evidence of a distinct peripheral inflammatory profile in sport-related concussion
Source: J Neuroinflammation. 2019 Jan 26;16:17. doi: 10.1186/s12974-019-1402-y (PMC6347801; doi:10.1186/s12974-019-1402-y)
Supplement: Supplementary file 3 — Table S3. PLSDA analysis of biomarker concentrations in healthy athletes dichotomized into two groups: ≤ 3 h from their last bout of physical activity vs. > 3 h from their last bout of physical activity. (DOCX 16 kb) [file 12974_2019_1402_MOESM3_ESM.docx]

**Additional file 3: Table S3.** Physical Activity and Biomarkers

| **Biomarker** | **PA within last 3h**  **(n = 15)** | **PA not within last 3h**  **(n = 75)** | **p value** | **FDR** |
| --- | --- | --- | --- | --- |
| IFN-γ | 3.7 (2.4 - 6.4) | 4.0 (3.2 - 5.1) | 0.44 | no |
| TNF-α | 1.7 (1.4 - 2.0) | 1.7 (1.5 – 1.9) | 0.90 | no |
| MPO (ng/mL) | 8.4 (6.2 - 12.6) | 11.9 (8.4 – 16.6) | 0.05 | no |
| IL-8 | 1.8 (1.3 - 2.4) | 2.1 (1.5 - 2.5) | 0.16 | no |
| Eotaxin | 80.6 (68.7 - 98.1) | 81.9 (67.4 – 90.8) | 0.92 | no |
| IP-10 | 168.7 (131.2 - 258.2) | 183.8 (154.8 – 240.0) | 0.32 | no |
| MCP-1 | 62.5 (54.6 - 73.4) | 68.2 (59.4 - 71.8) | 0.50 | no |
| MCP-4 | 20.0 (16.2 - 23.6) | 21.9 (19.2 - 26.8) | 0.19 | no |
| MIP-1β | 30.2 (25.4 - 39.5) | 32.8 (23.8 - 43.0) | 0.74 | no |
| TARC | 44.0 (34.5 - 58.9) | 63.4 (38.6 - 85.0) | 0.16 | no |

PA; physical activity; false discovery rate (FDR); interferon (IFN)-γ, tumor necrosis factor (TNF)-α, myeloperoxidase (MPO), interleukin (IL)- 8, eotaxin, interferon gamma-induced protein (IP)-10, monocyte chemoattractant protein (MCP)-1, -4, macrophage inflammatory protein (MIP)-1α, -1β, and thymus and activation-regulated chemokine (TARC).

All values reported as the median and interquartile range, in pg/mL unless otherwise stated.

P values are derived from bootstrap ratios, corrected at FDR < 0.05.

Athletes were dichotomized into two groups based on the time elapsed from their last bout of physical activity (< 3h; n = 15 vs. >3 h; n = 75), and differences in biomarkers between groups were analyzed by PLSDA. In this comparison, activity type consisted of games (n = 11, 12.2%), practice (n = 45, 50%), or exercise/workout (n = 29, 32.2%). Within the exercise/workout group, physical activity consisted of weight training (n = 15, 51.7%) cardiovascular training (n = 7, 24.1%) or a combination of both (n = 4, 13.8%). There were no significant differences in biomarker profiles between athletes who had their last physical activity session within the last 3 hours versus those who’s last session was >3 hours from the time of blood sampling.
