# Supplementary material for: Evidence of a distinct peripheral inflammatory profile in sport-related concussion
Source: J Neuroinflammation. 2019 Jan 26;16:17. doi: 10.1186/s12974-019-1402-y (PMC6347801; doi:10.1186/s12974-019-1402-y)
Supplement: Supplementary file 4 — Figure S1. PLS correlations between inflammatory profiles and days to blood draw post-injury in athletes with SRC. (DOCX 14 kb) [file 12974_2019_1402_MOESM4_ESM.docx]

**Additional file 4: Figure S1.** **Correlation between inflammatory profiles and days to blood draw post-injury.** interferon (IFN)-γ, tumor necrosis factor (TNF)-α, Myeloperoxidase (MPO), interleukin (IL)- 8, Eotaxin, interferon gamma-induced protein (IP)-10, monocyte chemoattractant protein (MCP)-1, -4, macrophage inflammatory protein (MIP)-1α, -1β, and thymus and activation-regulated chemokine (TARC). Plots show the correlation between blood biomarkers and days to blood draw post-injury in **A)** athletes with a sport-related concussion (SRC), and **B)** athletes with a musculoskeletal injury (MSK), by partial least squares (PLS) analysis. Bars represent biomarker loadings and the standard error derived from bootstrapped resampling (5000 samples). Red bars = significant correlation with days to recovery at a false discovery rate (FDR) <0.05.
