# Supplementary material for: Evidence of a distinct peripheral inflammatory profile in sport-related concussion
Source: J Neuroinflammation. 2019 Jan 26;16:17. doi: 10.1186/s12974-019-1402-y (PMC6347801; doi:10.1186/s12974-019-1402-y)
Supplement: Supplementary file 5 — Table S4. PLSDA analysis of biomarker concentrations in healthy male vs. female athletes. (DOCX 16 kb) [file 12974_2019_1402_MOESM5_ESM.docx]

**Additional file 5: Table S4.** Biomarker concentrations between males and females

| **Biomarker** | **Males (n = 54)** | **Females (n = 48)** | **P value** | **FDR** |
| --- | --- | --- | --- | --- |
| IFN-γ | 3.5 (2.4 - 4.6) | 3.9 (2.9 - 7.8) | 0.17 | no |
| TNF-α | 1.6 (1.4 - 1.9) | 1.6 (1.3 - 2.0) | 0.91 | no |
| MPO (ng/mL) | 11.1 (7.9 - 16.2) | 7.4 (5.8 - 11.0) | 0.00 | yes |
| IL-8 | 1.9 (1.6 - 2.5) | 1.6 (1.2 - 2.2) | 0.04 | no |
| Eotaxin | 84.9 (73.6 - 99.3) | 73.6 (63.4 - 94.1) | 0.01 | yes |
| IP-10 | 172.8 (130.2 - 230.0) | 159.3 (132.5 - 251.2) | 0.58 | no |
| MCP-1 | 68.8 (60.2 - 77.4) | 59.4 (50.9 - 69.0) | 0.00 | yes |
| MCP-4 | 21.5 (17.1 - 25.6) | 18.9 (16.7 - 22.6) | 0.13 | no |
| MIP-1β | 34.6 (26.4 - 42.7) | 27.7 (22.2 - 33.6) | 0.00 | yes |
| TARC | 48.3 (38.9 - 72.9) | 42.7 (32.7 - 56.9) | 007 | no |

false discovery rate (FDR); interferon (IFN)-γ, tumor necrosis factor (TNF)-α, myeloperoxidase (MPO), interleukin (IL)- 8, eotaxin, interferon gamma-induced protein (IP)-10, monocyte chemoattractant protein (MCP)-1, -4, macrophage inflammatory protein (MIP)-1α, -1β, and thymus and activation-regulated chemokine (TARC).

All values reported as the median and interquartile range, in pg/mL unless otherwise stated.

P values are derived from bootstrap ratios, corrected at FDR < 0.05.

PLSDA analysis of biomarker concentrations between male and female athletes showed significant differences. We observed significantly higher MPO (11.4 vs. 7.4 ng/mL) Eotaxin (84.9 vs 73.6 pg/mL), MCP-1 (68.8 vs. 59.4 pg/mL), and MIP-1β (34.6 vs. 27.7 pg/mL) blood concentrations in male vs. female athletes.
